# Supplementary material for: Transition to endemic: acceptance of additional COVID-19 vaccine doses among Canadian adults in a national cross-sectional survey
Source: BMC Public Health. 2022 Sep 14;22:1745. doi: 10.1186/s12889-022-14025-8 (PMC9473459; doi:10.1186/s12889-022-14025-8)
Supplement: Supplementary file 1 — Additional file 1: Appendix A-B. Table A1. Survey questions to determine Canadian adults’perceptions of additional COVID-19 vaccine doses and methods of delivery. Fig. B1. Influences on COVID-19 vaccine decision making by annual dose intention group. Fig. B2. Primary reason for previous COVID-19 vaccination by annual dose acceptance group. Fig. B3. Perception of COVID-19 vaccine co-administration with a) influenza vaccine, and b) routine vaccines, by annual dose intention group. Fig. B4. Preferred vaccination location by annual dose intention group. Fig. B5. Recommendations for making the vaccination process easier by annual dose intention group. [file 12889_2022_14025_MOESM1_ESM.docx]

**Appendix A**

**Table A1.** Survey questions to determine Canadian adults’ perceptions of additional COVID-19 vaccine doses and methods of delivery

| **Study variable** | **Survey question and response options** |
| --- | --- |
| **Questions asked of all survey respondents** | |
| Gender | **What is your gender? (Select all that apply)**  Woman, Man, Gender non-conforming, Transgender, Two-spirit, Not listed please specify |
| Region of residence | **In which province or territory do you live?**  British Columbia; Alberta; Saskatchewan; Manitoba; Ontario; Quebec; New Brunswick; Nova Scotia; Prince Edward Island; Newfoundland; Northwest Territories; Yukon; Nunavut |
| Self reported race and ethnicity | **What is your ethnic or cultural origin? (Select all that apply)**  White (e.g., Caucasian, European, etc.), Black (e.g., African, Haitian, Jamaican, etc.) Latin / Central American (e.g., Mexican, Colombian, Brazilian, Cuban, etc.), Arabic/West Asian/North African (e.g., Armenian, Egyptian, Iranian, Lebanese, Moroccan, etc.), East Asian (e.g., Chinese, Filipino, Japanese, Korean, Vietnamese, etc.), South Asian (e.g., Indian, Sri Lankan, etc.), Other, please specify, Prefer not to answer |
|  | **Do you self-identify as First Nations, Métis, or Inuk?**  Yes, No |
| First language | **What is the first language that you learned to speak?**  English, French, Other |
| Newcomer | **For respondents not born in Canada: When did you come to Canada?**  Between 2016 and 2020; Between 2011 and 2015; Before 2011 |
| Parent | **Are you the parent/primary guardian (e.g., birth parent, foster parent, stepparent, adoptive parent) who makes the health care decisions for one or more children 17 years old or younger?**  Yes, No |
| Disability | **Are you limited in the kind or amount of activity that you can do because of a long-term physical condition, mental condition, or health problem (sometimes referred to as 'a disability')?**  Yes, No, I don’t know, Prefer not to answer |
| Chronic illness | **To the best of your knowledge, do you currently have, or have you ever been diagnosed with any of the following diseases or conditions? (Yes to any one or more response options)**  Severe asthma requiring regular medical follow-up or hospitalization; Other severe chronic lung disease requiring regular medical follow-up or hospitalization (e.g., emphysema, chronic bronchitis, or cystic fibrosis); Severe heart problem requiring regular medical follow-up or hospitalization (e.g., angina, heart failure, heart attack); Diabetes; Liver disease; Chronic kidney disease; Cancer or another immune system disorder; Immunocompromised state (weakened immune system) from organ transplant or immune deficiencies; Obesity; Dementia |
| Healthcare worker | **Are you currently employed as a healthcare worker?**  Yes; No |
| Annual household income | **To the best of your knowledge, what is the total combined income before tax of everyone living in your household?**  $19,999 or less; Between $20,000 and $39,999; Between $40,000 and $59,999; Between $60,000 and $79,999; Between $80,000 and $99,999; Between $100,000 and $249,000; $250,000 or more; Prefer not to answer |
| Level of education | **What is the highest level of education you have completed?**  Some high school or less; High school diploma or equivalent; Registered Apprenticeship or other trades certificate or diploma; College, CEGEP or other non-university trade, certificate, or diploma; University certificate or diploma below bachelor's level; Bachelor's degree; Post graduate degree above bachelor's level; Prefer not to answer |
| Influenza vaccination intention | **Do you agree or disagree with the following: I plan to receive the flu vaccine this year (in Fall 2021 or Winter 2022)?**  Disagree; Neutral; Agree |
| COVID-19 disease history | **Have you had COVID-19 disease?**  Yes, confirmed by COVID-19 testing; I think so but it was not confirmed by COVID-19 testing; No; I don’t know; Prefer not to answer |
| COVID-19 vaccination status | **Have you received any doses of a COVID-19 vaccine?**  Yes; No |
|  | **If Yes to any doses: Have you received two doses of any COVID-19 vaccine?**  Yes; No |
|  | **Which of the following influenced your decision whether to get vaccinated against COVID-19? (Select all that apply)**  Conversations with friends and/or family; Messaging on social media; News reports; Health care professional recommendations (e.g., physician, nurse, pharmacist); Government/public health recommendations (e.g., National Advisory Committee on Immunization or public health officer); Personal and/or family health reasons; Other, please specify |
| **Questions asked of survey respondents who had received at least one dose of COVID-19 vaccine** | |
| COVID-19 dose 3 intentions | **If a third dose of COVID-19 vaccine was recommended, would you get it?**  Yes; No; I am undecided |
| COVID-19 annual dose intentions | **If it was recommended, would you get a COVID-19 vaccine every year (similar to the seasonal influenza vaccine)?**  Yes; No; I am undecided |
|  | **What is the main reason you got or would get a COVID-19 vaccine?∫**  It is/was mandated; To protect myself from COVID-19; To protect my family from COVID-19; To prevent the spread of COVID-19 in my community; To end the pandemic and return to normal life; Because it is recommended by experts and health care providers; Other, please specify |
|  | **If it was recommended, would you get the COVID-19 vaccine at the same time as the influenza vaccine?**  Yes; No; I am undecided |
|  | **If it was recommended, would you get the COVID-19 vaccine at the same time as a routine vaccine (e.g. tetanus or pneumococcal)?**  Yes; No; I am undecided |
|  | **What would be the easiest location for you to get the COVID-19 vaccine? (Select one)**  Doctor’s office; Pharmacy; Public health centre; A temporary vaccination centre; Place of employment; At my child’s school (answer for yourself, not your child); A mobile vaccination clinic/bus/van; Other, please specify |
|  | **What would make getting a COVID-19 vaccine easy for you? (Select all that apply)**  No appointment required (drop-in/walk-in clinic); COVID-19 vaccine services close to the community I live or work in; Transportation to/from vaccination clinics; Paid time off from work to get vaccinated; Vaccination information in the language I understand best; Culturally safe and welcoming vaccination settings; Allow my whole family to be vaccinated at the same time; Provide childcare funding/services while parents get vaccinated; Improve access to vaccination services for people with disabilities; Other, please specify; Prefer not to answer |

**Appendix B**


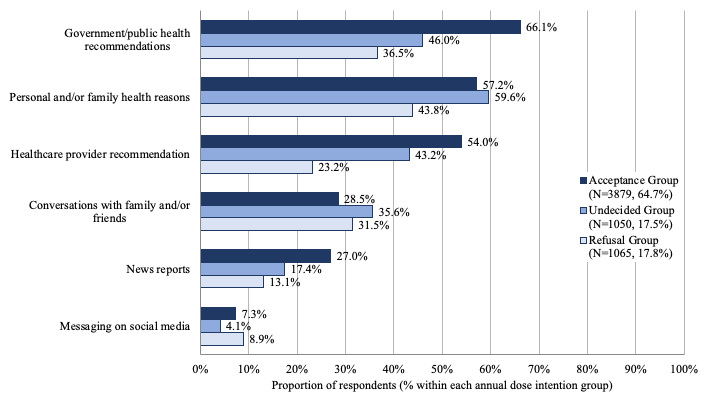


**Fig. B1.** Influences on COVID-19 vaccine decision making by annual dose intention group.

Respondents were asked to select all answers that apply.

**
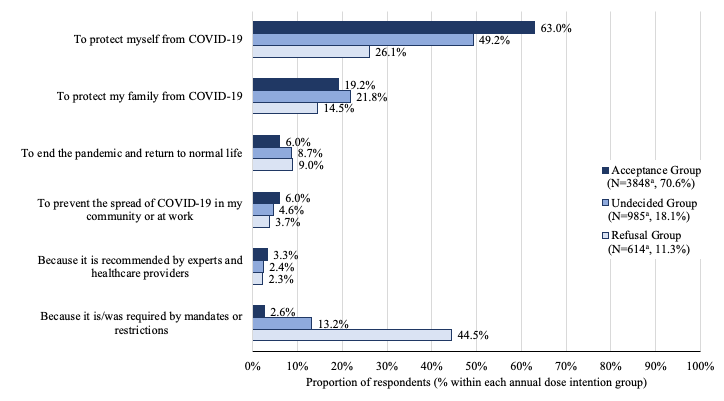
**

**Fig. B2.** Primary reason for previous COVID-19 vaccination by annual dose acceptance group.

^a^ Respondents who chose not to answer the question were removed from the denominator


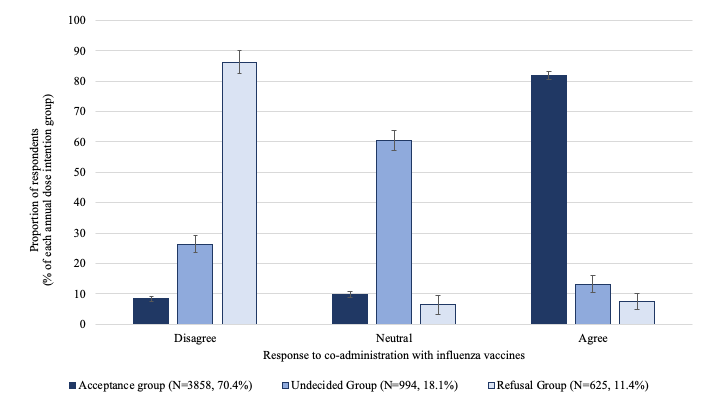


**a)**

**
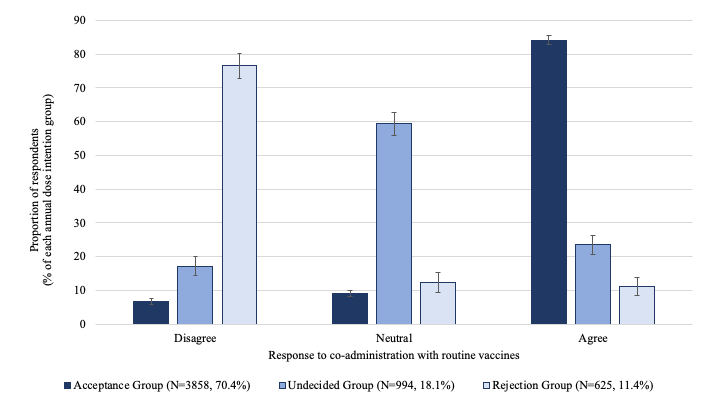
**

**b)**

**Fig. B3.** Perception of COVID-19 vaccine co-administration with a) influenza vaccine, and b) routine vaccines, by annual dose intention group.

Included only respondents who had received at least one dose of COVID-19 vaccine.


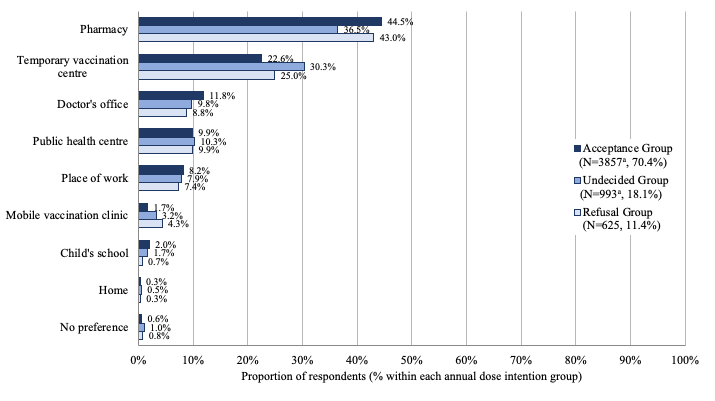


**Fig. B4.** Preferred vaccination location by annual dose intention group.

Included only respondents who had received at least one dose of COVID-19 vaccine. Respondents were asked to choose one answer.

^a^ One free-text response was discarded as not relevant to the question


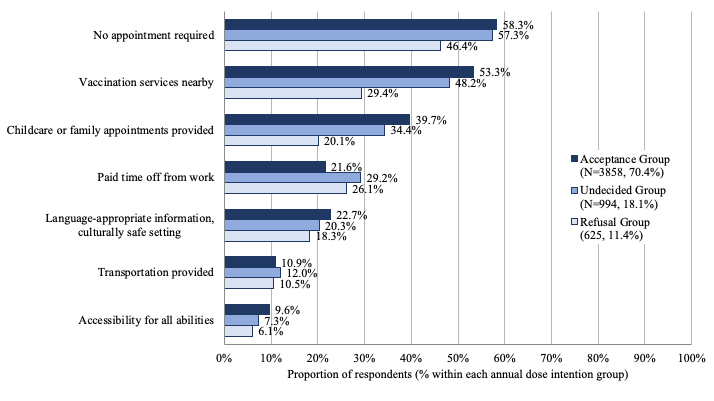


**Fig. B5.** Recommendations for making the vaccination process easier by annual dose intention group.

Included only respondents who had received at least one dose of COVID-19 vaccine. Respondents were asked to select all answers that applied.
